# A propos d’un cas de grossesse ovarienne

**DOI:** 10.11604/pamj.2016.25.175.10833

**Published:** 2016-11-18

**Authors:** David Kakez Nday, Ignace Bwana Kangulu, Léon Kabamba Ngombe, Jimmy Ngoie Nfundi, Gabriel Salumu, Patrick Nduwa Kameya, Michel Kabamba Nzaji, Henry Mundongo Tshamba

**Affiliations:** 1 Institut des Techniques Médicales de Kolwezi, République Démocratique du Congo; 2 Zone de Santé de Dilolo, Hôpital Général de Référence de Dilolo, République Démocratique du Congo; 3 Université de Kamina, Faculté de Médecine, Département de Santé Publique, République Démocratique du Congo; 4 Université de Lubumbashi, Faculté de Médecine, Département de Santé Publique, République Démocratique du Congo

**Keywords:** Grossesse ovarienne, échographie, consultations prénatales, Ovarian pregnancy, ultrasound, antenatal consultation

## Abstract

Nous rapportons un cas de grossesse ovarienne gauche découverte de manière passive en consultation externe à l'hôpital général de référence de Dilolo en République Démocratique du Congo. Le diagnostic a été confirmé à l'échographie, la prise en charge chirurgicale et les suites opératoires bonnes. La femme enceinte et le personnel médical devront être conscients de l'importance du bon suivi clinique et échographique de la grossesse pour le diagnostic précoce des implantations anormales.

## Abstract (en)

We here report a case of left ovarian pregnancy passively detected in the General Reference Hospital outpatient clinic in Dilolo, Democratic Republic of Congo. The diagnosis was confirmed by ultrasound; the patient underwent surgery with uneventful postoperative course. Pregnant women and healthcare personnel should be aware of the importance of proper clinical and echographic monitoring in pregnancy for early diagnosis of abnormal implantations.

## Introduction

La grossesse ovarienne est l´une des rares grossesses extra utérines avec un diagnostic souvent réalisé lors des interventions [1]. La grande proportion dans le timing de diagnostic se fait au troisième trimestre soit 45% [2], sa physiopathologie est mal connue, elle semblerait être secondaire à un reflux de l'ovocyte fécondé vers l'ovaire [3, 4]. Cependant plusieurs littératures mentionnent en cause à 57-90% des patientes sujettes de l'utilisation des stérilets comme le dispositif intra utérin [5]. Il existe des formes évolutives au-delà du 5^e^ mois, fréquentes dans les pays à faible densité médicale et exceptionnellement dans les pays développés [6–10]. Ce type de grossesses conduit facilement à des complications materno-fœtales par manque d'infrastructures sanitaires adaptées. La grossesse ovarienne, en République Démocratique du Congo(RDC), au vu des difficultés dans différents types d'accessibilités aux soins, reste une des pathologies rares au troisième trimestre malgré son diagnostic difficile réalisé en peropératoire, lors d'un examen échographique ou d'une analyse anatomo-pathologique [11]. Nous rapportons un cas de grossesse ovarienne observée à l'hôpital général de référence de Dilolo dans la province du Lualaba, territoire de Dilolo en RDC, cas reçu et traité au mois d'Avril 2016.

## Patient et observation

Madame H, âgée de 34 ans, 7^e^ geste et 6 enfants en vie, appartenant à une classe socio-économique basse, était venu consulter nos services pour inactivité fœtale après un périple et difficile voyage en février 2016. La patiente ne soulignait aucune fréquentation dans une structure sanitaire pour consultations en soins prénataux. L'état général de la patiente était conservé, la tension artérielle de 80/45 mm Hg, la température de 37,5^°^C et bonne coloration cutanéomuqueuse. L'abdomen était augmenté de volume, symétrique avec une Hauteur Utérine estimée à 26 cm, la présentation axiale utérine avec difficulté de palpation des pôles fœtaux et les bruits cardiaques fœtaux(BCF) absents à l'auscultation. A l´examen gynécologique, le col était long, ferme et fermé, un bombement du cul-de-sac de Douglass était noté. L´utérus était légèrement augmenté de volume. A la paraclinique, l'hémoglobine était dosée à 9g% et le bilan sanguin normal. L'échographie en premier lieu, moins précise, avait montré une grossesse normale avec le fœtus en intra utérin au vu de l'image confuse entre la paroi antérieure du sac et celle postérieure de l'utérus, tout en notant un vide observé dans le cul-de-sac où une liberté des mouvements d'anses faisait penser à une grossesse normale intra cavitaire avec mort fœtale. Après la maturation cervicale au Benzoate d'œstradiol, plusieurs tentatives de déclenchement du travail (Misopristol, Sonde de Folley en intra-cervical) se sont soldées en un échec.

**Figure 1:**
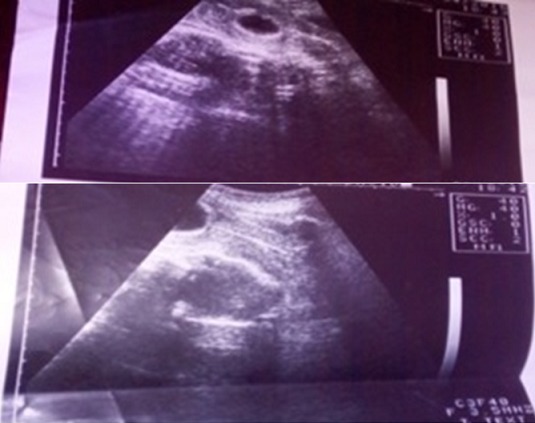
Image échographique d’un fœtus dans une coque en postérieur de l’utérus et une sonde en intra-utérin avec ballonné

**Figure 2:**
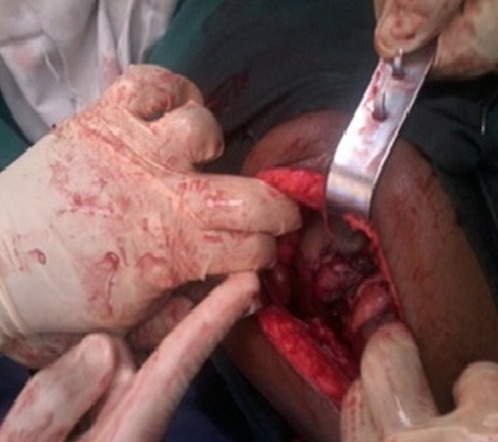
Image en peropératoire de la coque fixée à l’utérus en postérieur et montrant un vide entre la coque et les autres éléments abdominaux pelviens

**Figure 3:**
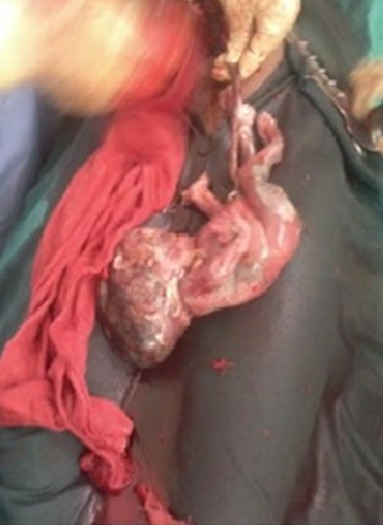
Fœtus macéré de sexe masculin pesant 1763 grammes issu de la grossesse ovarienne

Dans la suite d'hospitalisation, nous avons assisté deux fois à des chutes du taux d'hémoglobine (6g% et 6,5g%) qui avaient fait indiquer deux transfusions consécutives. Une seconde échographie réalisée avec la sonde ballonnée en place servant de repère (Figure 1) avait montré le ballonné en intra-utérin et un fœtus en céphalique avec peu de liquide amniotique dans une coque accolée en postérieur de l'utérus, adhérant aux annexes utérines. Au vu du tableau présenté, une laparotomie médiane sous ombilico-suspubienne était pratiquée sous anesthésie générale. Au cours de cette dernière, une coque au dépend des annexes gauches dont l'ovaire mais lié par un ligament de fixation s'accolant à l'utérus en postérieur et sans adhésions aux grêles, épiploons et annexes droits (Figure 2) était observé. L'ouverture de la coque hyper vascularisée dans la partie supérieure s'accolant au fond de l'utérus, après ligatures minutieuses des vaisseaux avait fait observer un fœtus fortement macéré de sexe masculin pesant 1763 grammes avec cordon ombilical s'arrachant au moindre mouvement et placenta inséré dans la partie supérieure presque à l'angle de jonction coque, partie restante de l'ovaire et fond utérin (Figure 3).

**Figure 4:**
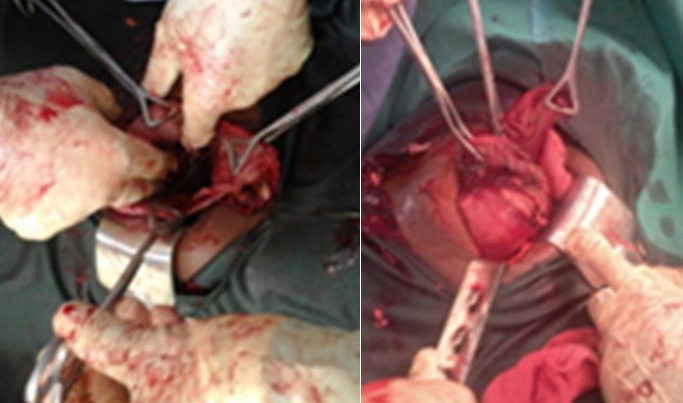
Ligature vaisseaux, incision coque et contrôle d’hémorragie

**Figure 5:**
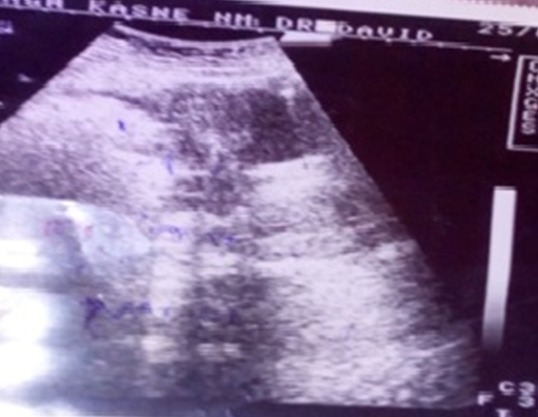
Image échographique au 32^e^ jour post-opératoire

De ces observations réalisées, a été faite une ligature sur la coque de part et d'autre du trajet des vaisseaux donnant jour aux berges incisés de la paroi abdominale pour manipulation et extraction facile fœtale , de cet acte s'en est suivi l'ouverture de la coque au bistouri froid ,extraction d'un fœtus de sexe masculin macéré suivi de celle du placenta avec hémostase, incision de la coque suivi de l'hémostase(Figure 4), nettoyage de la cavité abdominale au sérum physiologique , fermeture de la paroi abdominale plan par plan. Il s'en est suivi une bonne suite opératoire sans complications, le séjour était écourté au douzième jour, et le suivi échographique a montré un utérus normal (Figure 5).

## Discussion

### Fréquence

Des 5% des grossesses extra tubaires, la grossesse ovarienne prend à elle seule un taux de 2% de ces types de grossesses en dehors d'autres localisations rares [12]. La grossesse ovarienne reste un phénomène isolé exceptionnel dans la vie d'une femme en dehors des risques habituels et mécanismes exacts [1]. Elle est de diagnostic difficile dans nos milieux avec faible densité médicale, un taux faible d'accessibilité aux soins et le diagnostic est toujours tardif ainsi que la prise en charge difficile [2, 13–15]. Le taux le plus bas a été rapporté à Tunis : 1 sur 21439 naissances [2]. Cependant Chez nous, nous n'avons pas la fréquence nette disponible du moins les données historiques de notre hôpital général témoignent depuis sa création en 1958 un cas unique de grossesse ovarienne et abdominale sur une grossesse de 25 SA, sachant que la moyenne des naissances revient dans notre hôpital à 750 naissances environ l'an.

### Caractéristiques maternelles

Il s'agit d'un cas d'une patiente âgée de 34 ans, multi geste et multipare, de bas niveau socio-économique avec une grossesse qui n'était jusque-là suivi aux consultations prénatales. La littérature montre que l'âge des patientes varie entre 21 à 44 ans et la parité de 0 à 3 [2] et que l'âge avancé semble être associé à un risque accru de grossesses extra utérines suite à l'exposition durable aux facteurs de risque [16].

### Diagnostic

Sur le plan clinique et paraclinique, le diagnostic dépend que l'on soit au premier trimestre, deuxième ou troisième trimestre, sachant que la littérature parle de l'urgence pendant les quatre premiers mois de grossesse [15]. Plusieurs symptômes permettent d´orienter le diagnostic [17]: les troubles digestifs, les douleurs abdominales et pelviennes ,l´anémie avec altération de l'état général ,un fœtus très superficiel souvent en position atypique transversale haute ,une hémorragie interne ou extériorisée, ou un syndrome toxi-infectieux, au toucher vaginal, le col est souvent fixé ,dur et long sous la symphyse pubienne. Cependant dans notre cas, le diagnostic résulte d'un second examen échographique ayant objectivé un sac gestationnel en postérieur de l'utérus avec un fœtus inactif et sans battements cardiaques après échec de tentatives de déclenchement du travail. à cet âge-là au vu des difficultés dans le diagnostic échographique [18] aussi comme le dit Thoyer-Rozat qu'au-delà du cinquième mois il faudrait y penser pour reconnaitre la localisation extra utérine de la grossesse. La biologie peut montrer une anémie et une augmentation du taux de l´alpha-foeto-protéine [17], seul signe s'étant révélé en hospitalisation en dehors des nausées, vomissements et douleurs abdominales mis dans le compte des effets des produits utilisés en déclenchement du travail avec maturation cervicale (benzoate d'œstradiol) mais sans induction des contractions utérines [15]. Le diagnostic est souvent aussi confirmé en peropératoire [1], l'utérus est observé coiffé par le kyste fœtal ou coque [15] et aussi par analyse histologique ou anatomopathologique du tissu prélevé [11]. Cependant la description correspond à l'observation faite dans notre cas.

### Traitement et suites opératoires

Il est noté une rareté en ce qui concerne un traitement médical des grossesses ovariennes [19], traitement non encore élucidé et valable en fin premier trimestre et au-delà. La chirurgie est la thérapie utilisée dans la majorité des cas pour prendre en charge une grossesse ovarienne et dans les cas où la grossesse ovarienne est avancée en âge, l'ovariectomie, voir l'annexectomie peuvent se réaliser [1]. Mais une résection partielle de l'ovaire peut se faire afin d'assurer l'hémostase [18]. L´urgence dans les interventions tiens compte aussi de la viabilité fœtale [13]. La coque en tant que néoformation membraneuse doit être excisée après ligatures des grands vaisseaux et autres, le placenta extirpé tout en tenant compte de l'endroit de son insertion dans la coque. Dans notre cas, nous avons excisé la coque, enlever le placenta qui d'ailleurs avec le cordon ombilicale étaient en décomposition et un fœtus mort, fortement macéré. Une partie de l'ovaire a été sectionnée et l'hémostase assurée et les suites opératoires bonnes.

**Pronostic materno- fœtale** Il dépend en gros de la rapidité du diagnostic, l'attitude de prise en charge, la localisation de la grossesse, l'âge de la grossesse [15]. La morbidité maternelle est marquée par les complications opératoires hémorragiques [20], signes digestifs ou urinaires. La mortalité maternelle est de 0 à 18% [21]. Dans notre cas, la patiente a accusé deux séries d'anémies consécutives décompensées auxquelles la solution a été apportée. Pour le fœtus, on retrouve 40 à 60% des malformations [22], 75 à 95% voire 100% de mortalité périnatale [23]. Et dans notre cas le fœtus mort de sexe masculin, macéré ne présentait aucune malformation physique apparente.

## Conclusion

La grossesse ovarienne bien que rare, demeure une urgence obstétricale avec une séméiologie réservée et particulière dépendant des complications selon les périodes dans l'évolution de la grossesse. Son diagnostic reste difficile et se fait souvent en peropératoire, à travers l’échographie par une main expérimentée au vu des possibilités faibles que peut présenter la séméiologie. La prise en charge est chirurgicale. Le système sanitaire devra se faire doter du matériel et personnel technique compétent dans les structures et ainsi accroitre son accessibilité aux femmes enceintes, une possibilité de suivi et de prise en charge en cas d'urgence sur grossesse.
